# Engagement of SIRPα Inhibits Growth and Induces Programmed Cell Death in Acute Myeloid Leukemia Cells

**DOI:** 10.1371/journal.pone.0052143

**Published:** 2013-01-08

**Authors:** Mahban Irandoust, Julian Alvarez Zarate, Isabelle Hubeek, Ellen M. van Beek, Karin Schornagel, Aart J. F. Broekhuizen, Mercan Akyuz, Arjan A. van de Loosdrecht, Ruud Delwel, Peter J. Valk, Edwin Sonneveld, Pamela Kearns, Ursula Creutzig, Dirk Reinhardt, Eveline S. J. M. de Bont, Eva A. Coenen, Marry M. van den Heuvel-Eibrink, C. Michel Zwaan, Gertjan J. L. Kaspers, Jacqueline Cloos, Timo K. van den Berg

**Affiliations:** 1 Department of Pediatric Hematology/Oncology, VU University Medical Centre, Amsterdam, The Netherlands; 2 Department of Hematology, VU University Medical Centre, Amsterdam, The Netherlands; 3 Sanquin Research & Landsteiner Laboratory, Academic Medical Centre, University of Amsterdam, Amsterdam, The Netherlands; 4 Department of Hematology, Erasmus University Medical Center, Rotterdam, The Netherlands; 5 Dutch Childhood Oncology Group (DCOG), The Hague, The Netherlands; 6 School of Cancer Sciences, University of Birmingham, Birmingham, United Kingdom; 7 Department of Pediatric Hematology/Oncology, Medical School Hannover, Hannover, Germany; 8 University Medical Center Groningen, Groningen, The Netherlands; 9 Department of Pediatric Hematology/Oncology, Erasmus MC/Sophia Children's Hospital, Rotterdam, The Netherlands; Center for Cancer Research, National Cancer Institute, United States of America

## Abstract

**Background:**

Recent studies show the importance of interactions between CD47 expressed on acute myeloid leukemia (AML) cells and the inhibitory immunoreceptor, signal regulatory protein-alpha (SIRPα) on macrophages. Although AML cells express SIRPα, its function has not been investigated in these cells. In this study we aimed to determine the role of the SIRPα in acute myeloid leukemia.

**Design and Methods:**

We analyzed the expression of SIRPα, both on mRNA and protein level in AML patients and we further investigated whether the expression of SIRPα on two low SIRPα expressing AML cell lines could be upregulated upon differentiation of the cells. We determined the effect of chimeric SIRPα expression on tumor cell growth and programmed cell death by its triggering with an agonistic antibody in these cells. Moreover, we examined the efficacy of agonistic antibody in combination with established antileukemic drugs.

**Results:**

By microarray analysis of an extensive cohort of primary AML samples, we demonstrated that SIRPα is differentially expressed in AML subgroups and its expression level is dependent on differentiation stage, with high levels in FAB M4/M5 AML and low levels in FAB M0–M3. Interestingly, AML patients with high SIRPα expression had a poor prognosis. Our results also showed that SIRPα is upregulated upon differentiation of NB4 and Kasumi cells. In addition, triggering of SIRPα with an agonistic antibody in the cells stably expressing chimeric SIRPα, led to inhibition of growth and induction of programmed cell death. Finally, the SIRPα-derived signaling synergized with the activity of established antileukemic drugs.

**Conclusions:**

Our data indicate that triggering of SIRPα has antileukemic effect and may function as a potential therapeutic target in AML.

## Introduction

Currently only one third of adult patients diagnosed with acute myeloid leukemia (AML) can be cured despite aggressive chemotherapy, and relapse rate is still high in these patients [1], [2], [3]. Although the prognosis of pediatric AML patients is better, the outcome remains relatively poor. With standard induction chemotherapy, complete remission (CR) for newly diagnosed pediatric AML is achieved on more than 80% of patients, however, about 30–50% of these children relapse from minimal residual disease (MRD) cells that apparently survived chemotherapy [4], [5], [6]. Therefore, new treatment modalities for AML are warranted.

Distinct morphological subgroups in French-American-British (FAB) classification associate with different chromosomal rearrangements and acquisition of recurring genetic abnormalities; for example t(8;21))(q22;q22) and t(15;17))(q22;q21) create fusion genes, *AML/ETO* and *PML/RAR*α, which predominate in FAB M2 and M3 AML subtypes respectively. These proteins are two of the most common AML-associated oncofusion proteins, which in total represent 20% of the AML occurrence [7]. The cytogenetic rearrangement involved with t(8;21) disrupt genes that are required for normal hematopoietic development, such as subunits of core-binding factor [8]. Expression of the PML/RARα fusion protein leads to a differentiation block at the promyelocytic stage that can be relieved by all-*trans*-retinoic acid (ATRA). Studies on *AML/ETO* and *PML/RAR*α expressing cells have revealed that aberrant signaling pathways are involved [9]. Insights into these signaling pathways in AML at molecular level will pave ways for new treatment modalities.

Signal regulatory protein alpha (SIRPα) is a transmembrane receptor composed of 3 immunoglobulin-like domains in its extracellular region and an intracellular domain containing immunoreceptor tyrosine-based inhibitory motifs (ITIMs) which recruit and activate SHP-1 and SHP-2 [10], [11]. SIRPα is predominantly expressed on myeloid and neuronal cells [10] and its activation has been implicated in regulation of different cellular functions such as adhesion, migration, growth and differentiation [12], [13], [14]. Despite restricted expression of SIRPα, CD47, the natural ligand for SIRPα [15], is ubiquitously expressed and interacts with the SIRPα extracellular region. This interaction results in inhibition of phagocytosis by macrophages through tyrosine phosphatase activation and inhibition of myosin accumulation [16], [17], [18]. CD47 functions as a “don't eat me” signal and plays a key role in the programmed cell removal of abberant versus normal cells [19]. Indeed, it was recently shown that CD47 is overexpressed on AML leukemic stem cells as compared to their normal counterparts (hematopoietic stem cells) and this contributes to inhibition of phagocytosis and clearance of LSCs [20]. In addition, blocking antibodies directed against CD47 promoted phagocytosis as suggested through disruption of CD47-SIRPα interaction and this enhanced tumor clearance *in vivo* [20], [21], [22]. These findings are in line with several studies, which reported elimination of tumor cells by employment of CD47 blocking antibodies [20], [22], [23], [24], [25], [26], [27].

Although SIRPα is known to be expressed by AML cells as well [11], [28], its function on these cells has not been identified. Furthermore, previous studies were performed with antibodies that not only recognized SIRPα, but also several of the related molecules such as SIRPβ_1_ and SIRPγ. In the present study we have examined the mRNA and protein expression of SIRPα in a large cohort of AML patients and determined its relevance for AML cell survival. We show for the first time that SIRPα ligation triggers programmed cell death in AML cells and synergizes with antileukemic agents.

## Design and Methods

### Antibodies and drugs

At this moment no human agonistic SIRPα antibody is available. However, a rat agonistic SIRPα antibody (ED9) was generated in our laboratory [10], [29], that is also commercially available at Serotec (Oxford, UK). Such an agonistic antibody has much higher affinity to rat SIRPα as compared to CD47-Fc fragments [30], [31], so the use of this agonistic antibody was preferred for mechanistic studies and optimal SIRPα triggering. To be able to exert an agonistic signal using the available rat antibody, a chimeric construct of SIRPα was generated carrying rat SIRPα extracellular domain and human transmembrane and cytoplasmic region. The following monoclonal antibodies (mAb) were used in this study: ED9 (anti-rat SIRPα; mouse IgG1 isotype) was labeled with Alexa-633, which was obtained from Invitrogen (Breda, The Netherlands). Considering the differences between rat and human SIRPα, it is not likely that the ED9 agonistic antibody cross-reacts with the human SIRPα [30], [31]. For the experiments in the current study we used a concentration of 10 µg/ml ED9 antibody. This concentration may be considered as relatively high, but at this concentration the antibody is still specific since the negative controls (i.e. empty vector cells) show no response to this antibody at all. A dose-response curve is depicted in Figure S1.

Rabbit polyclonal Ab8120 (Abcam, Cambridge, United Kingdom) is directed to the cytoplasmic tail of human SIRPα. Mouse anti-actin monoclonal antibody, mAb1501R, was purchased from Chemicon International (Temecula, CA, USA). mAb against caspase-3 was obtained from Cell Signaling Technology (Boston, MA, USA). APC-labeled anti-human CD11b was acquired from BD pharmingen (San Jose, CA, USA). PE- labeled B6H12 was purchased from Santa Cruz Biotechnology and B6H12 F(ab')2-fragments were generated in our laboratory by pepsin digestion as previously described [32].

The histone deacetylase (HDAC) inhibitors (Trichostatin A (TSA), valproic acid (VPA) and sodium butyrate) and ATRA were purchased from Sigma Aldrich (St Louis, MO, USA). 5-aza-2-deoxycytidine (DAC, decitabine) was kindly provided by Pharmachemie BV (Haarlem, The Netherlands). Cytarabine (Cytosar®) was obtained from Pharmacia & Upjohn (Woerden, The Netherlands). Daunorubicin (Cerubidine®) was purchased from Rhone Poulenc Rorer (Amstelveen, The Netherlands). Etoposide (PV16, Vepesid®) was obtained from Bristol-Myers Squib (Woerden, The Netherlands). Imatinib was provided by Novartis (The Netherlands). zVAD was obtained from Merk Biosciences (Darmstadt, Germany).

### Patient samples

For the expression array experiments bone marrow and/or peripheral blood samples were collected from adult AML patients at diagnosis, as described by Valk PJ et al. [33]. Bone marrow and/or peripheral blood samples from children diagnosed with *de novo* AML were collected from the following study centers: VU University Medical Center, Amsterdam, The Netherlands; The Dutch Childhood Oncology Group (DCOG), The Hague, The Netherlands and the AML BFM-study Group, Hannover, Germany. AML subtypes were classified according to the criteria by Bennett *et al*., including the modifications to diagnose FAB subtypes [34]. Mononuclear cells were isolated by density gradient centrifugation as described previously [35]. All samples contained at least 80% leukemic cells, as determined morphologically by analyzing May-Grünwald-Giemsa (Merck, Darmstadt, Germany) stained cytospins.

### Cell Lines and culture conditions

The human leukemic cell lines KG1a (primitive human hematopoietic myeloid progenitor), Kasumi-1 (human acute myeloid leukemia, FAB M2 t(8;21)), HL-60 (human promyelocytic leukemia), NB4 (human acute promyeloctic leukemia, FAB M3 t(15;17)),U937 (human acute monocytic leukemia), THP-1 (human acute monocytic leukemia), CEM (human acute lymphoblastic leukemia), Jurkat (human T-cell acute lymphoblastic leukemia) were routinely cultured in RPMI 1640 medium (Gibco Laboratories, Irvine, UK) supplemented with 10% fetal calf serum (Integro BV, Dieren, the Netherlands). Kasumi-1 cells (0.5×10^6^) were incubated for 0, 3, 24, 48, 72 and 96 hours with 1 µM DAC, 0.3 µM TSA, 0.5 mM VPA and 1 mM sodium butyrate in 5% CO_2_ humidified air at 37°C. Cells were subsequently used for Western Blot analysis, as described below. Human neutrophils were isolated from heparinized blood of healthy individuals by centrifugation over isotonic Percoll (Pharmacia, Uppsala, Sweden) and subsequent lysis of erythrocytes as described [36]. Neutrophils were cultured in Hepes-buffered saline solution supplemented with 1% human serum albumin (Cealb: Sanquin, Amsterdam, the Netherlands) and 5 mM glucose.

### DNA isolation

#### Cell lines

1–5×10^6^ of KG1a, Kasumi-1 and HL-60 cells were resuspended in HIRT buffer (0.6% SDS, 10 mM Tris, 10 mM EDTA pH 8). Proteinase K was added and samples were incubated at 50°C for 2 hours and subsequently at 37°C overnight. Phenol: chloroform (1∶1) was added and the solution was mixed vigorously and centrifuged. The aqueous layer was transferred into a tube and the phenol/chloroform extraction was repeated. Following centrifugation, the aqueous layer was removed and the DNA was precipitated by NaOAC/EtOH (1∶24), washed with 70% EtOH and resuspended in TE buffer. DNA concentrations were measured with spectrophotometer (Nanodrop, Isogen, The Netherlands).

#### Patient samples

DNA was isolated from cryopreserved cytospins. A sterile swab and a drop of sterile water were used to wipe the cells from the slides. The swab was transferred into buffer (100 mM Tris, 10 mM NaCl, 5 mM EDTA, 1% SDS pH 9) containing proteinase K and incubated overnight at 52°C. Tubes were centrifuged and DNA was isolated as described above. For the method of the bisulphate sequencing of the DNA see Methods S1.

### Western blot analysis

Cells were washed in PBS, centrifuged and the cell pellet was lysed with Igepal lysis buffer (Sigma-Aldrich) containing protease inhibitor cocktail (Roche). Whole cell lysates were clarified by centrifugation and denatured in Laemmli's sample buffer (Bio-rad Laboratories, Hercules, CA, USA). After that cell lysates were subjected to Western blot and membranes stained with primary and secondary antibodies.

### Construction of retroviral vectors and transduction of Kasumi-1 cells

The rat-human SIRPα fusion construct (chSIRPα) was generated from cDNA and PCR fragments as follows: nucleotide 1–1236 of the rat SIRPα cDNA [10] was fused to nucleotide 1230–1509 of the human cDNA (prot. accession No: NM_080792). The chSIRPα protein contains amino acids 1–412 (rat extracellular domain) and amino acids 411–503 (human transmembrane and cytoplasmic region) resulting in a total length of 505 amino acids, including the signal sequence. The sequence of the construct was confirmed by automated sequencing.

For retroviral transduction the chSIRPα construct was cloned into the retroviral expression vector pLZRSpMBN-linker-IRES-eGFP(NotI) [37]. The Phoenix-A packaging cell line [38] was transfected with the retroviral construct containing chSIRPα or empty vector (EV) containing eGFP by calcium-phosphate transfection. After puromycin selection (Sigma-Aldrich, St Louis, MO, USA), harvested virus supernatant [37] was used for transduction of Kasumi-1 or NB4 cells. chSIRPα-expressing cells were subsequently selected in several rounds by FACS sorting (MoFlo,Dako Cytomation) on the basis of eGFP expression to reach to >98% positive cells. Cell surface expression of chSIRPα was determined by FACS analysis using the ED9 mAb, as described below. FACS-sorted, mock-transduced cells containing EV were used as controls in all experiments. Ectopic expression of SIRPα and/or eGFP were regularly monitored and were found to be stable for several months, with >90% positive cells.

### Cell proliferation and programmed cell death

For cell proliferation assays cells were seeded in triplicate at 1×10^5^/ml concentration in 96-well plates and treated with 10 µg/ml of the ED9 mAb where indicated and incubated for the mentioned time points up to 7 days. For programmed cell death (PCD) experiments, cells were seeded at 0.5×10^6^ cells/ml and on days 1 and 3 PCD was measured by Annexin V-phycoerythrin and 7-amino-actinomycin D (7-AAD) or DAPI double staining according to the manufacturer's protocol (BD Pharmingen, San Jose, CA, USA). All analyses were performed on a FACS calibur (BD Biosciences, San Jose, CA, USA). Cells positively stained with Annexin-V and 7-AAD negative were considered to be early apoptotic.

### Growth inhibition studies

Growth inhibitory effects of chemotherapeutics in combination with ED9 mAb were evaluated with the MTT-assay, as described previously [39]. SIRPα and EV cells were incubated with 4 concentrations of cytarabine (ara-C) 2.5–0.002 µM, daunorubicin (DNR) 18.0–0.005 µM, etoposide (VP16) 4.4–0.01 µM and imatinib 5−0.005 µM in combination with one fixed concentration of the ED9 mAb (10 µg/ml); these experiments were done in triplicate. Within each experiment all drugs were tested alone, as well as in combination. Drug interactions between chemotherapeutic drugs and ED9 mAb were studied by using the multiple drug effect analysis of Chou and Talalay [40] (Calcusyn software, Biosoft, Cambridge, UK) and antagonistic, additive or synergistic interactions were determined. This method is commonly used in many drug interaction studies [41]. The interactions are determined by Combination Index (CI) which indicates synergism (CI<0.9), additivity (CI = 0.9–1.1) or antagonism (CI>1.1). In the CI-FA plot the CI values >0.5 are evaluated and per experiment a mean CI was calculated from FA values 0.5, 0.75 and 0.9. The average CI (± SD) of three experiments is given for each of the combinations.

### Statistical analysis

The statistical significance of measured differences in proliferation and PCD between the various conditions and cell populations was determined using the paired Student's t-test. Calculations were performed using Graph-pad Prism and SPSS software. Statistical analysis (Cox proportional hazards model; reported p values corresponded to the Wald test) on overall survival and event free survival was performed in SPSS software. Survival distribution was compared with median SIRPα expression of the whole group.

## Results

### SIRPα mRNA expression in AML

**Figure 1:**
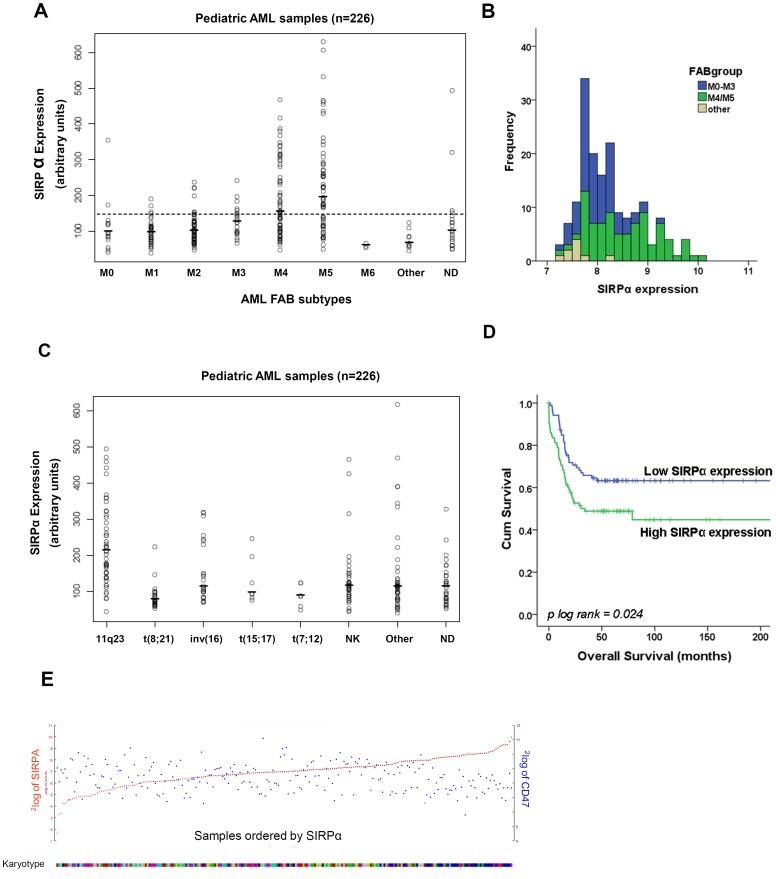
SIRPα mRNA expression and its prognostic effect in pediatric AML cohort. (A) SIRPα mRNA expression was determined in different FAB subtypes. The dots represent individual patients and the horizontal bar is the group mean. The horizontal dotted line represents the mean levels (139.6; n = 5) of SIRPα expression in normal CD34+ HSC. (ND: not determined). (B) Frequency of SIRPα expression among different FAB subtypes of AML patients is shown as stacked histograms. (C) SIRPα mRNA expression as stratified after karyotype (NK: normal karyotype). (D) Overall survival of pediatric AML patients (n = 175) stratified according to either low (< median of 8.1) or high (≥ median 8.1) SIRPα mRNA expression. (E) Correlation between CD47 and SIRPα mRNA expression is shown by the blue and red dots, respectively. The lower bar demonstrates the cluster of patients in karyotypes, in which higher SIRPα expression is clustered in blue, representing the MLL rearrangement group on the right side.

The mRNA level in pediatric AML patients was analyzed in a micro-array dataset containing 226 samples [42]. SIRPα mRNA expression varied considerably among different AML patients (Figure 1A). A clear association was observed between SIRPα expression and AML FAB subtypes with the highest levels found in the myelo-monocytic FAB M4/M5 subsets. The SIRPα expression levels in myeloblastic leukemic blasts were relatively low in the FAB M0–M3 subtypes. The M6 erythroid type of AML also showed a low SIRPα expression. Comparing acute myeloid leukemia cases with normal bone marrow, lower expression of SIRPα was observed in immature AML subtypes (Figure 1A), suggesting a myeloid differentiation stage-dependent expression, which is in line with the high expression of SIRPα found on normal monocytes and macrophages [10]. In particular, the significant difference between M0–M3 and M4/M5 (p<0.001), and the different expression distribution are depicted in Figure 1B. Classifying pediatric samples based on karyotypes showed the highest (p = 1.63×10^−10^) and lowest (p = 2.05×10^−6^) expression of SIRPα in MLL-rearranged and t(8;21) subgroups, respectively (Figure 1C). Since we only accessed one pediatric dataset, we validated these findings in adult AML datasets [33], [43], [44] and consistent with the pediatric results the highest level of SIRPα expression was found in M4 and M5 subtypes as compared to the immature groups such as FAB M0, M1, M2 and M3 (Figure S2). In addition, karyotype classification of 285 AML patients in an adult dataset [33], showed increased SIRPα expression in inv(16), and MLL-rearrangement groups (depicted as clusters 5, 9 and 16) in comparison to t(8;21) and t(15;17) AML (Figure S1B).

To examine whether SIRPα expression is correlated with patient survival, we performed an analysis on overall survival (OS) and event free survival (EFS) in the pediatric cohort (n = 175), for which follow-up data are available. We observed that higher SIRPα expression compared to the median of the 175 patients (8.1 arbitrary units), significantly correlated with unfavorable outcome. Figure 1D shows the Kaplan Meier analysis based on OS (log-rank p = 0.024, hazard ratio (HR) 1.7, p = 0.026). For EFS the data were similar: log-rank p = 0.029, and HR: 1.5, p = 0.031. In addition, following stratification on karyotype, we could not generally find any significant relation between SIRPα and outcome (not shown). Only within the MLL rearranged samples a high SIRPα expression (above the median SIRPα expression of the MLL rearranged group) exhibited a trend towards unfavorable outcome (HR: 2.28, p = 0.062).

It is difficult to compare the outcome between children and adults since the adults have, in general, a very dismal prognosis. In the data set of Valk et al. [30] no association was found between SIRPα expression and outcome on the whole cohort. For the adult MLL rearranged samples, high SIRPα levels associated with a slightly favorable outcome (HR: 0.84, p = 0.027). In both children and adults, multivariate analysis reveals that SIRPα is not an independent risk factor.

Recent studies have shown that CD47, the ligand for SIRPα, is a prognostic factor in breast cancer and its expression correlates with SIRPα expression in bone marrow and peripheral blood of breast cancer patients [32], [45]. We therefore evaluated a possible association between SIRPα and CD47 expression in different datasets [33], [42], [46], however we did not find any evidence for such an association in AML. Figure 1E shows that CD47 is equally distributed among the pediatric AML subtypes while there is a clear difference in SIRPα expression with the MLL-rearranged clustering in the high SIRPα range.

### SIRPα protein expression in AML

**Figure 2:**
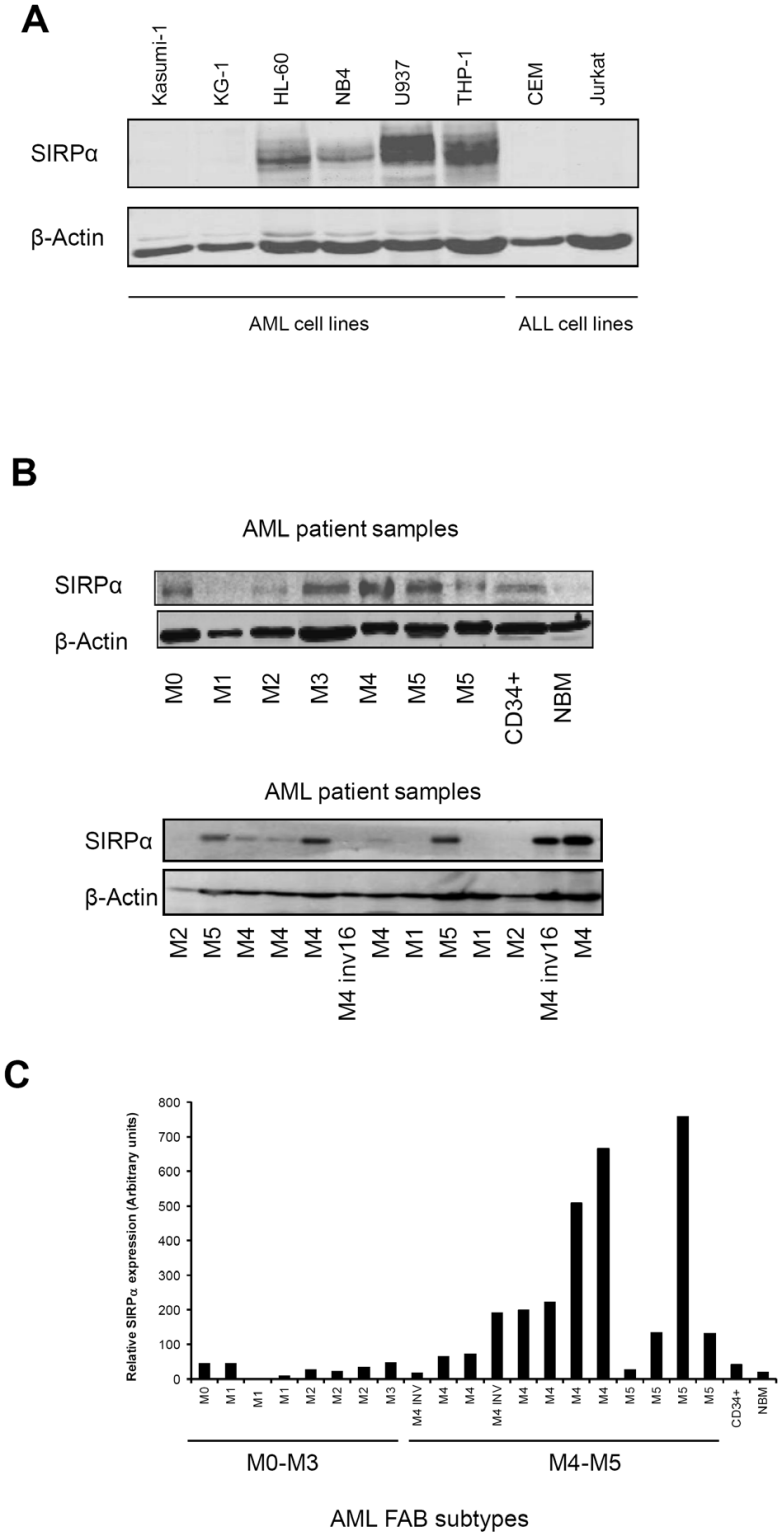
SIRPα protein expression in AML cell lines and patients. Western blot analysis was performed in (A) cell lines and (B) 20 pediatric AML patient samples. β-actin staining was used as loading control. (C) SIRPα expression is quantified relative to β-actin expression.

We determined SIRPα protein expression, by Western blotting using an antibody directed to the cytoplasmic tail of the human SIRPα on various leukemic cell lines and patient samples. While no expression was observed among acute lymphoblastic leukemia (ALL) cell lines, AML cell lines differentially expressed the SIRPα protein (Figure 2A). In particular, immature myeloblasts such as t(8;21) Kasumi-1, KG-1, HL60 cells or promyelocytes like t(15;17) NB4 cells expressed low or undetectable levels of SIRPα protein compared to more differentiated monocytic cells such as THP-1 and U937 (Figure 2A). We also analyzed 20 primary pediatric AML patient samples and consistent with the mRNA data, SIRPα protein expression was low/undetectable in immature subgroups compared to the more mature groups such as M4 and M5 (Figure 2B, C). As expected we did not observe SIRPα expression in ALL patient samples (n = 10) (Figure S3). Collectively, these findings suggest a selective myeloid and differentiation stage-dependent expression of SIRPα mRNA and protein expression in AML.

### Upregulation of SIRPα upon differentiation of t(15;17) AML cells

**Figure 3:**
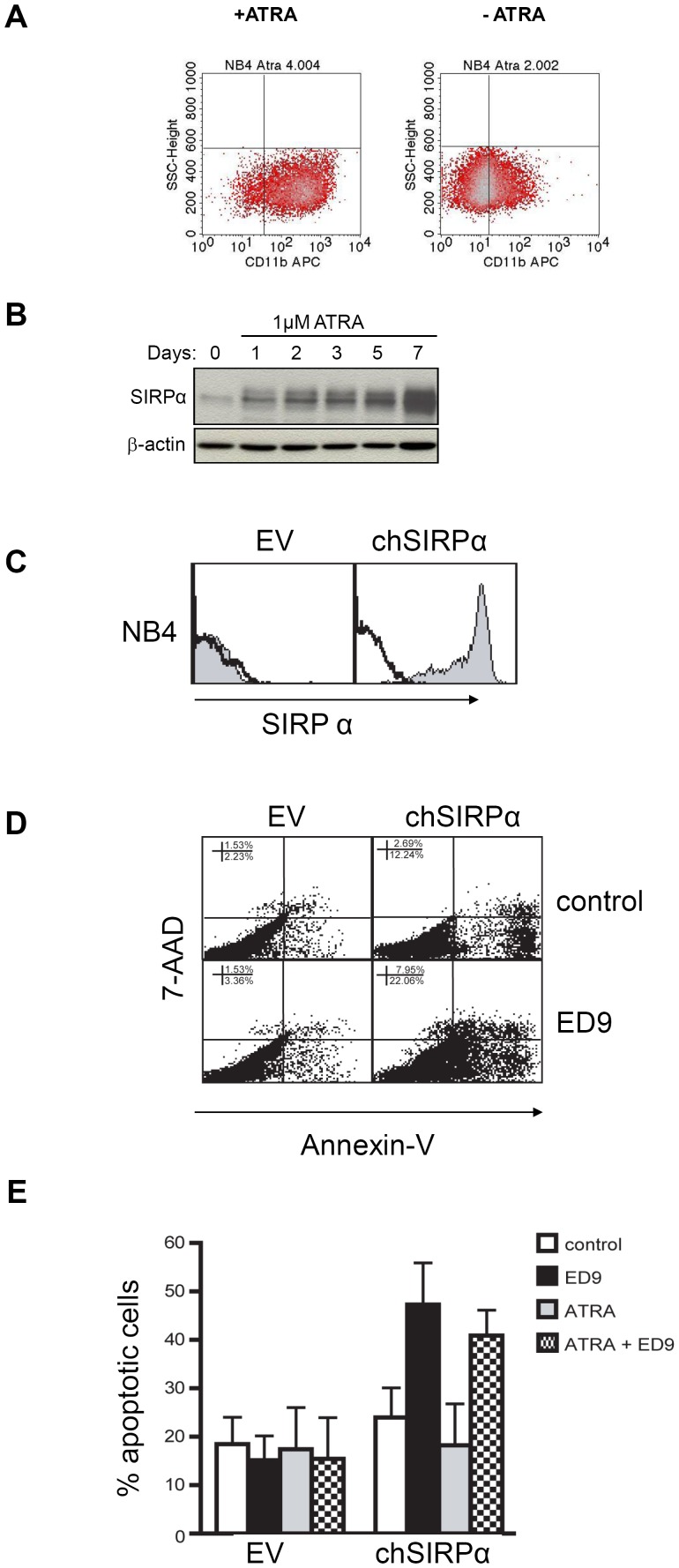
Upregulation of SIRPα upon differentiation of t(15;17) NB4 cells and induction of cell death following its triggering. (A) NB4 cells were exposed to 1 µM ATRA and granulocytic differentiation of the cells was examined by cell surface expression of the common myeloid marker, CD11b. (B) SIRPα protein expression, determined by western blotting, is upregulated in ATRA-incubated NB4 cells. β-actin is used as a loading control. (C) Flow cytometric analysis of chSIRPα surface expression is determined by using ED9 mAb in transduced NB4 empty vector and chSIRPα expressing cells. (D) 24 hrs following ED9 (10 µg/ml) incubation, the percentage of cell death in chSIRPα and EV transduced NB4 cells was quantified by APC-Annexin V and PE-7AAD FACS staining. (E) Percentage of apoptosis after exposure to 1 µM ATRA is shown in combination with 10 µg/ml of ED9.

To address whether SIRPα expression is upregulated upon differentiation of AML cells, we selected the NB4 cell line, a t(15;17) M3 FAB subtype, which only express low levels of SIRPα (Figure 2A). Since ATRA treatment of t(15;17) APL patients is known to result in granulocytic differentiation [47], [48], we examined if SIRPα expression increased after exposure to ATRA. To address this, the NB4 cells were incubated with 1 µM ATRA for 7 consecutive days and granulocytic differentiation of the NB4 cells was confirmed by upregulation of the common myeloid marker CD11b (Figure 3A). In concert with the increased differentiation of NB4 cells, SIRPα protein expression was markedly upregulated following ATRA exposure (Figure 3B). This upregulation was already detectable after 24 hrs and it was further increased during the following days of treatment.

### Induction of programmed cell death in t(15;17) AML cells following SIRPα ligation

Our initial experiments with the rat myeloid cell line NR8383 showed that several agonistic monoclonal antibodies [49] against rat SIRPα (for example ED9, ED17 or OX41) or recombinant Fc-fusion proteins containing the extracellular region of CD47, the natural ligand of SIRPα, are able to suppress cell proliferation (Figure S4). In order to investigate the effect of SIRPα ligation in NB4 cells we tested a variety of previously reported antibodies against SIRPα, but these either lacked the appropriate specificity, showing cross-reactivity with other SIRP family members, or lacked the agonistic activity [28], [32]. In addition CD47-Fc did not show a sufficiently high affinity for binding to SIRPα in our *in vitro* experiments to be used as an agonistic (data not shown). Hence, to be able to study the effect of SIRPα triggering in human myeloid cells, we generated a chimeric SIRPα (chSIRPα) construct that enabled the use of the rat specific SIRPα agonistic ED9 mAb. This chSIRPα construct consisted of the extracellular region of rat SIRPα and the transmembrane and the cytoplasmic domains of human SIRPα [10]. Stable t(15;17) NB4 cell lines expressing chSIRPα or empty vector (EV) were generated by retroviral transduction. Flow cytrometric analysis of the retrovirally transduced and FACS-sorted cells showed that the vast majority (>90%) of cells had been transfected by chSIRPα (Figure 3C). The levels of chSIRPα expression (i.e. mean fluorescence) were comparable to those seen, with the same mAb, on rat macrophages or granulocytes ([10] and data not shown).

Ligation of SIRPα in NB4 cells by agonistic ED9 mAb resulted in induction of programmed cell death (PCD) as quantified by flow cytometry using annexin-V/7-AAD staining (Figure 3C). After 24 h of exposure to ED9 mAb, the percentage of annexin-V positive cells was significantly higher in the NB4 chSIRPα cells (47.3±8.6%), as compared to NB4 EV cells (15.1±5.0%; p = 0.009) (Figure 3C). These data support the requirement for ED9 binding to SIRPα, since no induction of cell death was observed in NB4 EV cells. These findings provide evidence for induction of cell death capacity by SIRPα triggering in APL cells.

From a therapeutic perspective, it would appear beneficial to relieve differentiation block in M3 AML cells by using ATRA and as a result to upregulate SIRPα expression, which can subsequently be targeted by an agonistic antibody to induce PCD. Clearly prerequisite for such a strategy to be successful would be the efficiency of SIRPα triggering following differentiation. Therefore we examined whether apoptosis induction via SIRPα persisted after differentiation with ATRA. Stably transduced NB4 (chSIRPα and EV) cells were exposed to 1 µM ATRA in combination with the fixed concentration of 10 µg/ml ED9 mAb, which was shown to trigger PCD in the NB4 chSIRPα cells. As expected, ATRA treatment alone did not have any effect on PCD, whereas ED9 resulted in PCD in the chSIRPα transduced NB4 cells and this was not significantly altered after differentiation with ATRA. Furthermore while SIRPα triggering by ED9 mAb does induce programmed cell death, we found that it did not affect differentiation (Figure S5).

Taken together, these data indicate that ATRA provides a stimulus for differentiation of t(15;17) APL cells and this results in upregulation of SIRPα expression to a level that makes the cells prone to cell death induction via SIRPα triggering even in differentiated M3 cells.

### Upregulation of SIRPα following differentiation of t(8;21) AML cells

To examine whether the low endogenous expression of SIRPα is also upregulated following differentiation of other low-SIRPα-expressing myeloid leukemic cells, we selected t(8;21) Kasumi-1 cells. These cells belong to AML M2 FAB subtype and express low endogenous levels of SIRPα (Figure 2A). It has been shown that histone deacetylase (H-DAC)-inhibitors such as butyrate, valproic acid (VPA) and trichostatin (TSA) and DNA methyltransferase (DNMT)- inhibitors such as decitabine, induce granulocytic maturation of t(8;21) acute myeloid leukemia cells [50], [51], [52], [53] (and data not shown).

**Figure 4:**
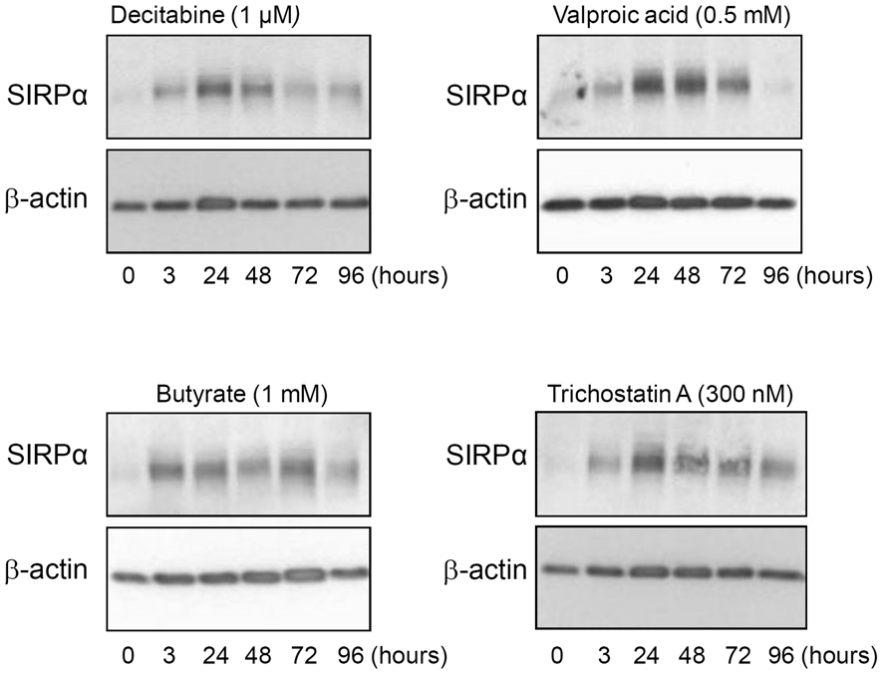
SIRPα upregulation in t(8;21) Kasumi-1 cells following treatment with inhibitors of epigenetic gene silencing. Kasumi-1 cells were incubated with 1 µM Decitabine, 0.5 mM valproic acid, 1 mM Butyrate and 300 nM Trichostatin. Endogenous SIRPα protein level, determined by Western blotting was upregulated at indicated time points. β-actin staining was used as a loading control.

Kasumi-1 cells were exposed to TSA, VPA,butyrate or decitabine for indicated time points, which resulted in markedly increased SIRPα levels (Figure 4). With all drugs, an increase in SIRPα protein expression was detected as early as 3 hrs after exposure and this expression reached maximal levels after approximately 24 hrs. These data show that SIRPα is upregulated following differentiation of Kasumi-1 cells.

An alternative explanation for the upregulation of SIRPα in t(8;21) AML was that these inhibitors of epigenetic silencing had acted directly on the SIRPα gene (accession number: NP-542970.1), and in fact this seemed possible since a prominent CpG island is present in the *PTPNS1* promoter region. DNA methylation in this region was explored in Kasumi-1 cells and four t(8;21) AML patients by bisulphite DNA sequencing. Results revealed actually very low levels of DNA methylation in the promoter region (Figure S6). We also analyzed the *SIRPA2p* pseudogene, in which abundant methylation was detected in the corresponding region [49]. Taken together these data strongly suggest that the increased level of SIRPα in t(8;21) Kasumi-1 cells following demethylating agents is the result of differentiation.

### Inhibition of proliferation and induction of PCD in t(8;21) AML cells by SIRPa triggering

**Figure 5:**
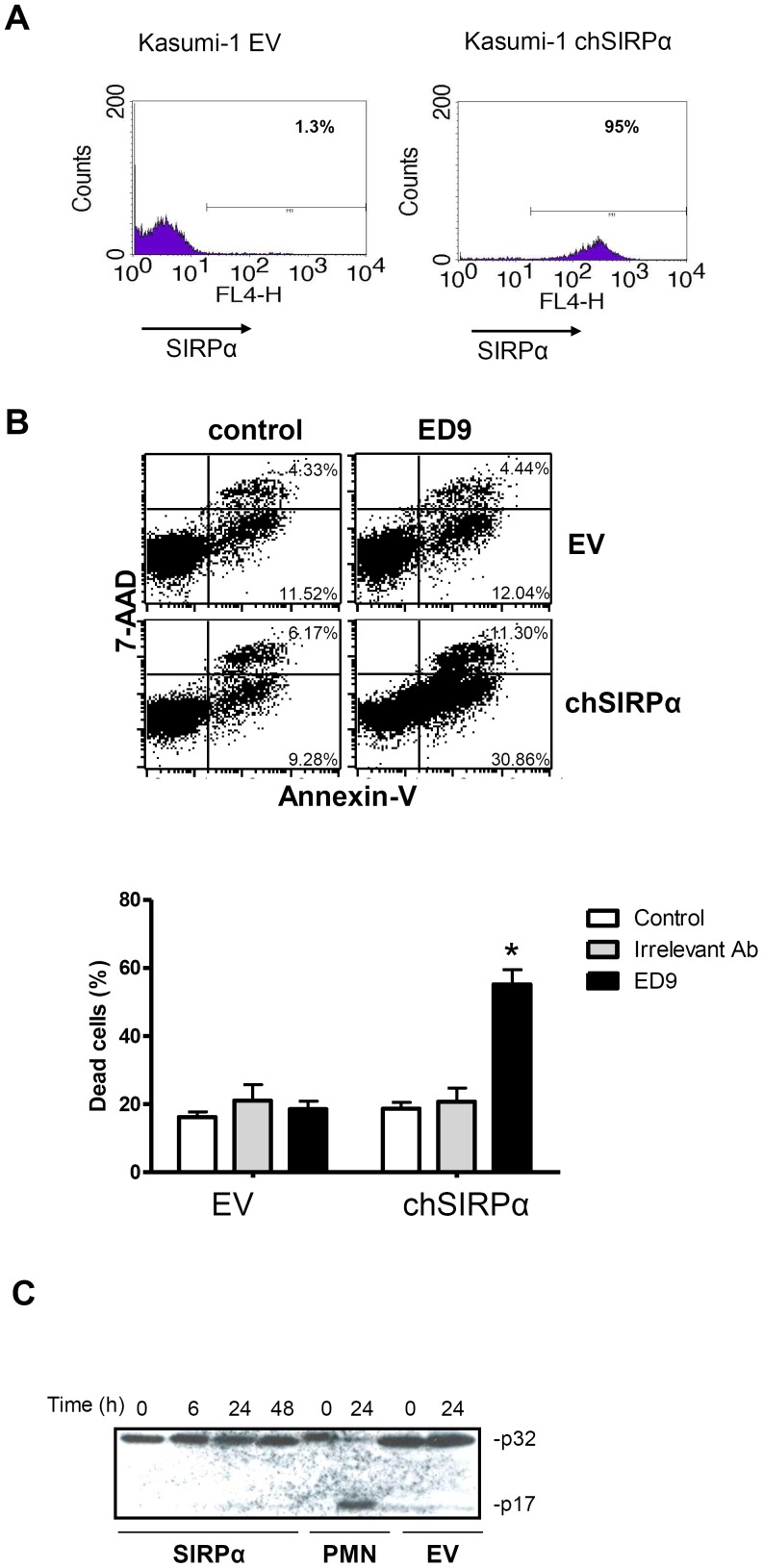
Ligation of chSIRPα induces caspase 3-independent PCD in Kasumi-1 cells. (A) Flow cytometric analysis of SIRPα expression was performed by using ED9 mAb in stable Kasumi-1 cells expressing chSIRPα and EV. (B) Kasumi-1 chSIRPα and EV cells were incubated with 10 µg/ml ED9 mAb and the percentage of cell death was determined after 24 hrs. Annexin V and 7-AAD FACS staining defined that ligation of chSIRPα resulted in increased cell death in chSIRPα Kasumi-1 cells compared to EV control cells. Data are means ± SD calculated from 3 independent experiments using triplicate samples (*: significant difference *p*<0.05). (C) Kasumi-1 cells expressing chSIRPα or EV were treated with 10 µg/ml ED9 for mentioned time points. Caspase 3 staining shows no cleavage of the p32 subunit. As a positive control for caspase 3 cleavage, human neutrophils (PMN) were incubated at room temperature for 0 and 24 hours.

Since triggering SIRPα by agonistic ED9 mAb induced cell death in t(15;17) NB4 cells, we extended our findings in t(8;21) Kasumi-1 cells, stably transduced with chSIRPα or EV (Figure 5A). The overexpression of chSIRPα in Kasumi-1 cells itself did not affect the growth, however culturing Kasumi-1 chSIRPα cells in the presence of the agonistic rat ED9 antibody caused a significant inhibition of proliferation (Figure S7). In order to investigate whether growth suppression triggered by chSIRPα ligation coincided with an enhanced level of cell death induction, the percentage of cell death was quantified by flow cytometry, using Annexin V and 7-AAD staining (Figure 5B). Already 24 hours after adding ED9 mAb the percentage of early dying cells, defined as Annexin V positive, was significantly higher in the Kasumi-1 chSIRPα cells, as compared to Kasumi-1 EV cells, while addition of an irrelevant antibody had no effect (Figure 5B, lower panel). Similar results were observed on day 3 of treatment, at which ligation with ED9 mAb had caused significant cell death in Kasumi-1 chSIRPα cells (35.2±15.3% versus 10.4±2.8% in untreated control cells; *p* = 0.02). All effects required ED9 binding to chSIRPα, since no induction of cell death was observed in Kasumi-1 EV cells.

We next investigated whether cell death induction in Kasumi-1 cells was involved caspase activity, which is often required for PCD. First, we investigated activation of the effector caspase-3, which can be measured by evaluating the appearance of the p17 caspase-3 cleavage product. As shown in Figure 5C triggering of SIRPα in Kasumi-1 chSIRPα cells did not result in any detectable caspase-3 cleavage, whereas this was detected upon culture of freshly isolated neutrophils. Furthermore, incubation with the universal inhibitor of caspases, zVAD (10 µM), did not affect programmed cell death induction via SIRPα in Kasumi-1 chSIRPα cells whereas neutrophil apoptosis was zVAD sensitive (results not shown). Collectively, these findings indicate a growth-suppressive and caspase-independent mode of PCD induction via SIRPα in t(8;21) AML.

To examine whether the observed effects of ED9 mAb in Kasumi-1 cells, occur through blocking of CD47-SIRPα interactions, we used the blocking anti-CD47 antibody B6H12. As shown in Figure S8, induction of cell death by ED9 cannot be mimicked by B6H12. This experiment shows at least that the pro-apoptotic/growth regulatory effects that we report in this study are not simply due to a blocking of cis or trans CD47-SIRPα interactions and are more likely due to agonism of the ED9 antibody that was actually also reported by us before in another context [54].

### SIRPα ligation synergizes with conventional antileukemic and targeted agents in t(8;21) and t(15;17) AML cells

**Figure 6:**
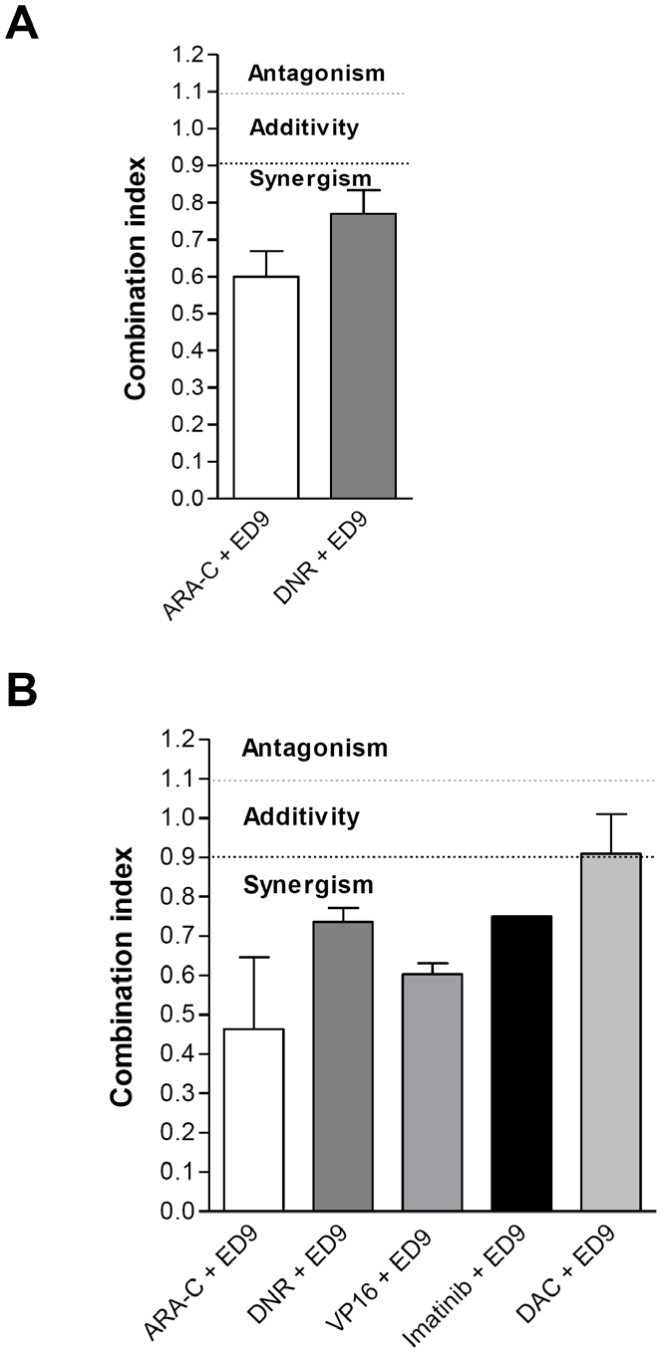
SIRPα-derived signal synergizes with different antileukemic drugs. Inhibition of cell growth is depicted by combination of ED9 mAb (10 µg/ml) with (A) Ara-C and DNR in NB4 cells expressing chSIRPα (B) Ara-C, DNR, VP16, DAC and imatinib in Kasumi-1 cells expressing chSIRPα. Results are based on means of 3 experiments and are calculated using Calcusyn.

Considering the potential of exploiting SIRPα targeting to improve the treatment of AML patients, we examined the efficacy of the ED9 mAb in combination with clinically relevant chemotherapeutic agents used for the treatment of AML. NB4 chSIRPα and EV cells were exposed to cytarabine (ARA-C) and daunorubicin (DNR) in combination with ED9 mAb. Survival was monitored after 4 days using a range of chemotherapeutic drug concentrations, which had shown appropriate dose response curves in pilot experiments. We used a fixed concentration of ED9 mAb (10 µg/ml), which had been shown to promote PCD in NB4 cells (Figure 6A). Co-incubation of NB4 cells stably expressing chSIRPα with each of the two chemotherapeutics and ED9 mAb resulted in synergistic effects (combination indexes (CI) using standard calcusyn calculation were CI_DNR_ = 0.0.59±0.04 and CI_ARA-C_ = 0.60±0.2). No effect of ED9 mAb was seen in the NB4 EV cells (data not shown).

In addition, Kasumi-1 chSIRPα and EV cells were exposed to ARA-C, DNR, and VP16 in combination with ED9. Since the Kasumi-1 cell line has been described to harbor an activating c-kit mutation [55], we also tested ED9 in combination with the tyrosine kinase inhibitor imatinib mesylate (Figure 6B). Similar to the experiments with the NB4 cell line, this was tested using a 4-day survival assay applying a range of drug concentrations, which had shown appropriate dose response curves in pilot experiments, and a fixed concentration of ED9 mAb (10 µg/ml). The leukemic cell survival of Kasumi-1 EV and chSIRPα cells after incubation with ED9 alone were 98±2.4% and 87±4.4%, respectively. Co-incubation of Kasumi-1 SIRPα cells with each of the four anti-leukemic drugs and ED9 resulted in synergism which was indicated by a shift in the dose-response curve. By using the standard Calcusyn calculations for combination effects, a synergistic effect of ED9 incubation was observed for all applied chemotherapeutic drugs (combination indexes (CI) include: CI_ARA-C_ = 0.46±0.32; CI_DNR_ = 0.74±0.06, CI_VP16_ = 0.55±0.066 and CI_Imatinib_ = 0.75±0.11) (Figure 6B).

Taken together, these results show that expression and ligation of chSIRPα provide growth inhibitory effects in t(15;17) and t(8;21) AML cells and this had a synergism with established anti-leukemic drugs.

## Discussion

Insights into the molecular pathogenesis of AML have paved the way for new treatment strategies that specifically target gene products implicated in induction of the leukemia. In the present study we have investigated the role of SIRPα as a potential target in the treatment of AML. By evaluating SIRPα mRNA levels in both pediatric and adult cohorts of AML patients, we observed a differential expression of SIRPα in AML subtypes. Interestingly, high expression of SIRPα was observed in more mature AML subgroups (M4 and M5) in comparison to immature subtypes, normal bone marrow and CD34+ blast cells. There was interpatient vairability of SIRPα expression between patients with M4 or M5 subtypes, which is probably due to heterogeneity of AML. Nevertheless, higher expression in mature subtypes is consistent with a differentiation related expression of SIRPα. Consistent with this, an upregulation of SIRPα was also observed during differentiation of AML cell lines, which express low endogenous levels of SIRPα.

The expression of SIRPα was not correlated to the expression of its ligand CD47, which was ubiquitously expressed on the AML blasts. This is consistent with a recent study performed by Nagahara et al. who showed that increased expression of SIRPα and CD47 was not correlated on breast cancer cell lines. However a stronger correlation was observed in the bone marrow and peripheral blood of breast cancer patients compared to normal cases [45]. They suggest that such host factor characteristics may have implications for prognosis of breast cancer. We also evaluated mRNA levels of other related genes in the various datasets of AML. No clear association was observed between CD47 and SIRPα or different FAB subtypes or karyotypes. In our AML study, the association between SIRPα and prognosis is not strong and does not function as an independent factor. Galbaugh et al showed that SIRPα mRNA was high in triple negative breast cancer and related to an increased invasiveness of the tumor [56]. However, thus far there are no publications that show a clear correlation between SIRPα expression and outcome, even for breast cancer. The lack of differential expression of CD47 in our study might be due to the fact that we investigated the bulk of the AML cells and not the leukemic stem cells, for which it was previously established that CD47expression is associated with poor prognosis [20].

We demonstrate here for the first time, that SIRPα ligation using agonistic monoclonal antibodies inhibits cell growth and promotes cell death induction. Our results suggest that PCD via SIRPα is caspase-independent. This mechanism of cell death induction has been reported previously in the context of AML [57], in which no involvement of caspases in cell death of an AML cell line was observed upon treatment with chemotherapeutic drugs. It should be noted that CD47 ligation also induces caspase-independent PCD [57], and it would seem reasonable to assume that this was due to a potential blocking of *cis*-interactions between CD47 and SIRPα on AML, rather than to agonistic triggering of both receptors. However, observations show that this was not likely to be the case, since the effects on t(15;17) NB4 cells and t(8;21) Kasumi-1 cells could not be reproduced using blocking antibodies against CD47 (Alvarez et al, not shown). The actual mechanism underlying the pro-apoptotic effect of SIRPα triggering is under investigation. Clearly, one obvious candidate to mediate growth inhibitory signals, particularly in myeloid cells, is the cytosolic tyrosine phosphatase SHP-1. Our preliminary findings show that SHP-1 and also the related SHP-2 are in fact abundantly expressed in fresh AML samples and in established AML cell lines (data not shown). In rodent macrophage cell lines the hematopoietic phosphatase SHP-1 can indeed be recruited and activated upon triggering of SIRPα by its natural ligand CD47, as well as by the agonistic antibody ED9 used herein [54].

It should be emphasized that while the survival and proliferation of AML may not be directly regulated by CD47-SIRPα interactions, there the *in vivo* life span of leukemic cells may well be affected by them in another way. In particular, recent studies have demonstrated that CD47 can act as an anti-phagocytic or so called “don't eat me” signal that prevents clearance of human leukemic cells by macrophages in xenogeneic mouse models *in vivo* [20], [21]. Other “don't eat me” signals such as CD200 have also been shown to be upregulated in multiple tumors including AML [58]. Leukemic stem cells appear to have higher levels of CD47 than normal CD34+ HSC cells and this could provide them with a selective advantage for survival. In this study we show that other signals such as SIRPα can be upregulated to encourage such don't eat me signals and subsequent evasion from programmed cell removal [19], [58]. It must be noted that CD47 signaling can also be regulated through binding to its ligand, thrombospondin-1(TSP-1). This CD47-TSP-1 interaction has been shown to inhibit response to nitric oxide and correspondingly increase radiosensitivity. As a result, blocking such interactions could confer therapeutic radioprotection of normal tissues [59], [60], [61].

Analysis of AML patients had revealed that higher levels of CD47 are associated with a poor prognosis [17], [18]. While the results of these studies indicate that blocking of the interaction between CD47 and SIRPα may be of interest from a therapeutic perspective, our current results suggest that it may, perhaps even simultaneously be beneficial to trigger SIRPα as well. Especially since targeting the CD47–SIRPα phagocytic pathway alone is likely to have toxic effects [58]. In fact, the ED9 antibody against SIRPα that we have used herein appears both capable of triggering programmed cell death as well as to block CD47-SIRPα interaction [62]. Clearly, future studies are needed to generate the suitable agents that can trigger apoptosis via human SIRPα and validate it as a potential treatment target in AML.

## Supporting Information

Figure S1 **Dose-response curve of ED9 antibody induced apoptosis in Kasumi-1 cells.** EV = kasumi cells tranduced with empty vector, WT = kasumi cells tranduced with wild type SIRPα. Apoptosis was measured after exposure to a range of ED9 antibody concentrations. 10 µg/ml was selected as optimal concentration for further studies. At this concentration no effect was seen on cells only expressing the (human) constitutive SIRPα.
(PPT)

Figure S2 **SIRPα mRNA expression in adult AML cohort.** (A) SIRPα mRNA expression was determined in different FAB subtypes of 285 adult patients. The dots represent individual patients and the horizontal bar is the mean of the group (ND: not determined). (B) Adapted correlation view of the 16 unsupervised clusters (indicated on the left) of 285 adult AML specimens identified by mRNA profiling [33], including the expression levels of SIRPα using 3 independent probes on the right diagonal axes. SIRPα expression is high in clusters 5, 9 and 16, but low in most other clusters, including clusters 12 and 13, which contain almost exclusively t(15;17) and t(8;21) AML, respectively.
(PPT)

Figure S3 **SIRPα is not expressed in ALL patient samples.** Analysis of protein expression of SIRPα in pediatric ALL patient samples by western blotting showed that SIRPα is not expressed in these samples. β-actin staining was used as a loading control.
(PPT)

Figure S4 **Triggering SIRPα in the rat NR8383 macrophage cell line inhibits proliferation.** NR8383 cells were incubated for 18 hours with CD47-Fc protein or indicated anti-rat SIRPα monoclonal antibodies (ED9, ED17 or OX41). ^3^H-thymidine was added for 4 hours and proliferation was determined by incorporated radioactivity.
(PPT)

Figure S5 **NB4 cells differentiate by ATRA exposure.** Differentiation of NB4 cells stably expressing chSIRPα and EV was examined by flow cytometry after treatment with ATRA or ED9. increased expression of CD11b was observed only after ATRA but not by ED9 treatment.
(PPT)

Figure S6 ***PTPNS1*** **promoter region is not methylated.** Each circle indicates a CpG dinucleotide (open circles: unmethylated, filled circles: methylated) and each line represents analyses of a single amplified clone. *SIRPα2p* pseudogene, which is highly highly homologous to *PTPNS1* was used as a positive control with high degree of methylation [49]. Methylation specific PCR and bisulphate sequencing [63] of the Kasumi-1 cell line and four t(8;21) AML patients did not reveal methylation of the *PTPNS1* promoter region.
(PPT)

Figure S7 **SIRPα ligation results in inhibition of proliferation in Kasumi-1 cells.** Kasumi-1 cells expressing chSIRPα or EV, were incubated with ED9 mAb for 7 days and cell proliferation was evaluated by daily cell counting. Data are means ± SD calculated from 3 independent experiments using triplicate samples.
(PPT)

Figure S8 **Blocking anti-CD47 antibody cannot mimic ED9 effects in Kasumi-1 cells.** (A) Flow cytometry data of DAPI and Annexin-V staining and (B) Summary graph illustrates the quantified flow cytometric data. Kasumi-1 cells expressing chSIRPα or EV were incubated with ED9 mAb or B6H12 as blocking anti-CD47 antibody. Percentage of cell death was increased significantly in the case of ED9 treatment compared to EV but B6H12 anti-CD47 incubation did not have this effect.
(PPT)

Methods S1 Detailed method description of the DNA bisulphate sequencing.
(DOC)
