# Supplementary figures and images for: Engagement of SIRPα Inhibits Growth and Induces Programmed Cell Death in Acute Myeloid Leukemia Cells
Source: PLoS One. 2013 Jan 8;8(1):e52143. doi: 10.1371/journal.pone.0052143 (PMC3540026; doi:10.1371/journal.pone.0052143)

## Slide 1
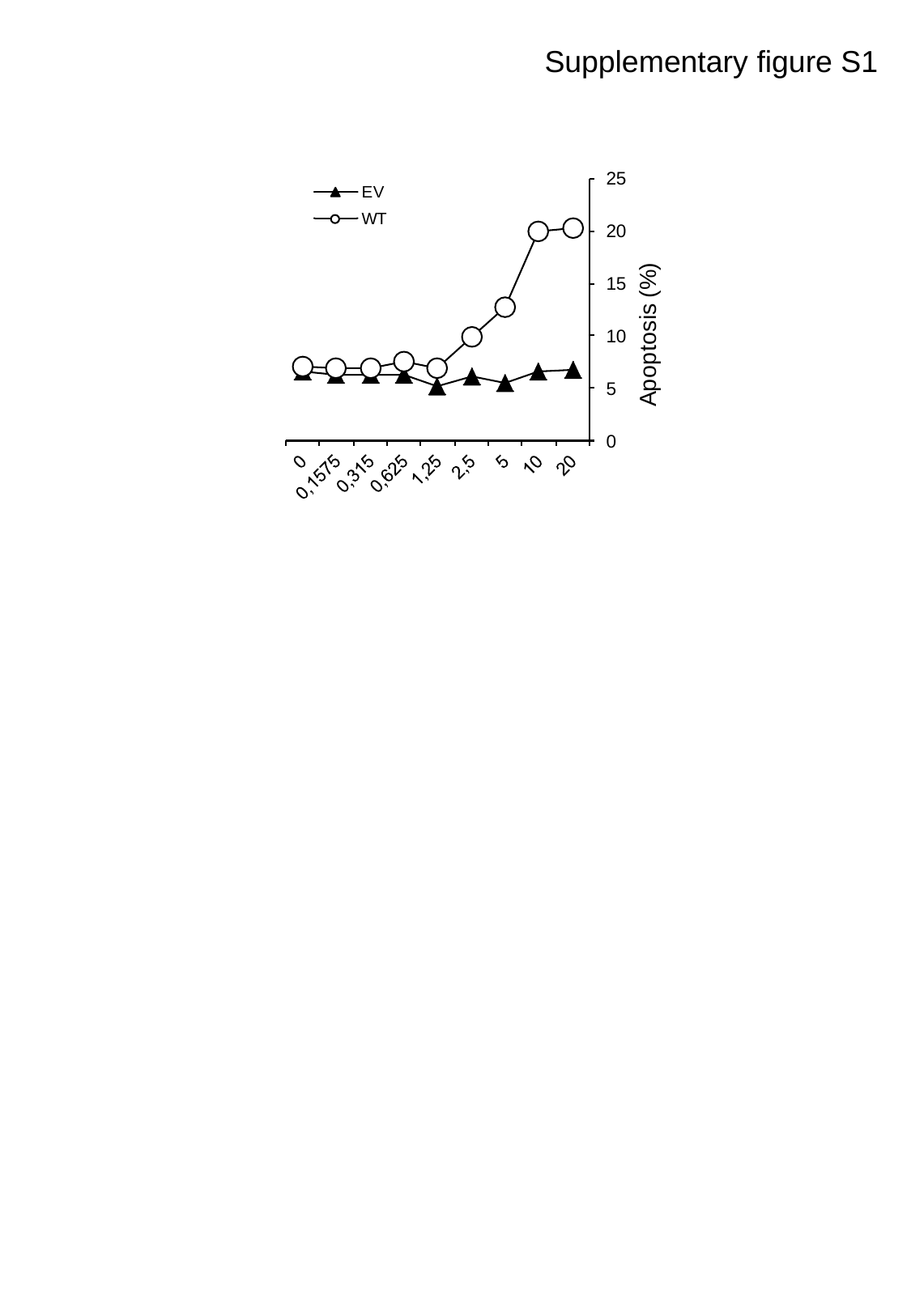

Supplementary figure S1

Supplement: Figure S1 — Dose-response curve of ED9 antibody induced apoptosis in Kasumi-1 cells. EV = kasumi cells tranduced with empty vector, WT = kasumi cells tranduced with wild type SIRPα. Apoptosis was measured after exposure to a range of ED9 antibody concentrations. 10 µg/ml was selected as optimal concentration for further studies. At this concentration no effect was seen on cells only expressing the (human) constitutive SIRPα. (PPT) [file pone.0052143.s001.ppt]

## Slide 1
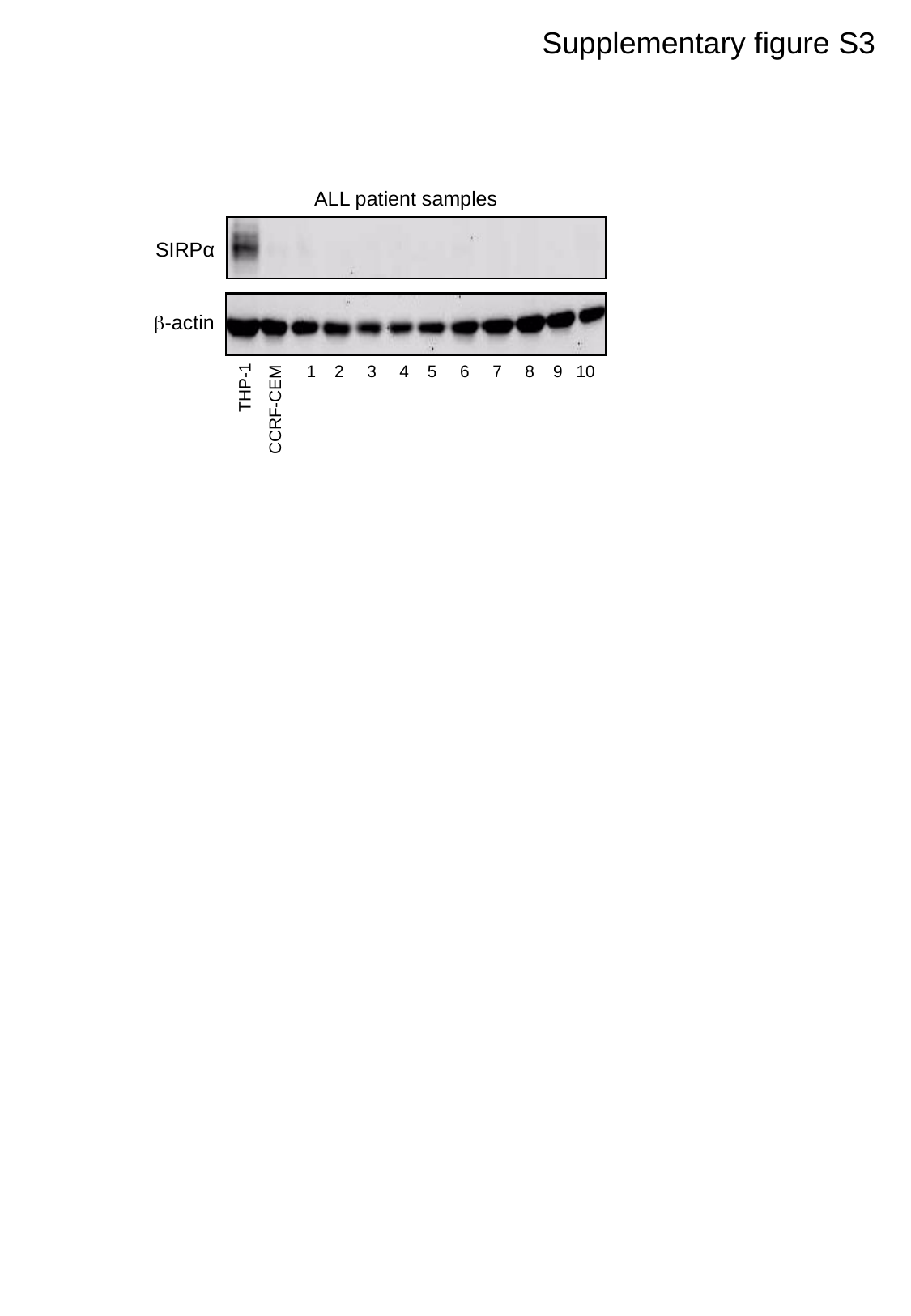

Supplementary figure S3
ALL patient samples
SIRPα
β-actin
1 2 3 4 5 6 7 8 9 10
THP-1
CCRF-CEM

Supplement: Figure S3 — SIRPα is not expressed in ALL patient samples. Analysis of protein expression of SIRPα in pediatric ALL patient samples by western blotting showed that SIRPα is not expressed in these samples. β-actin staining was used as a loading control. (PPT) [file pone.0052143.s003.ppt]

## Slide 1
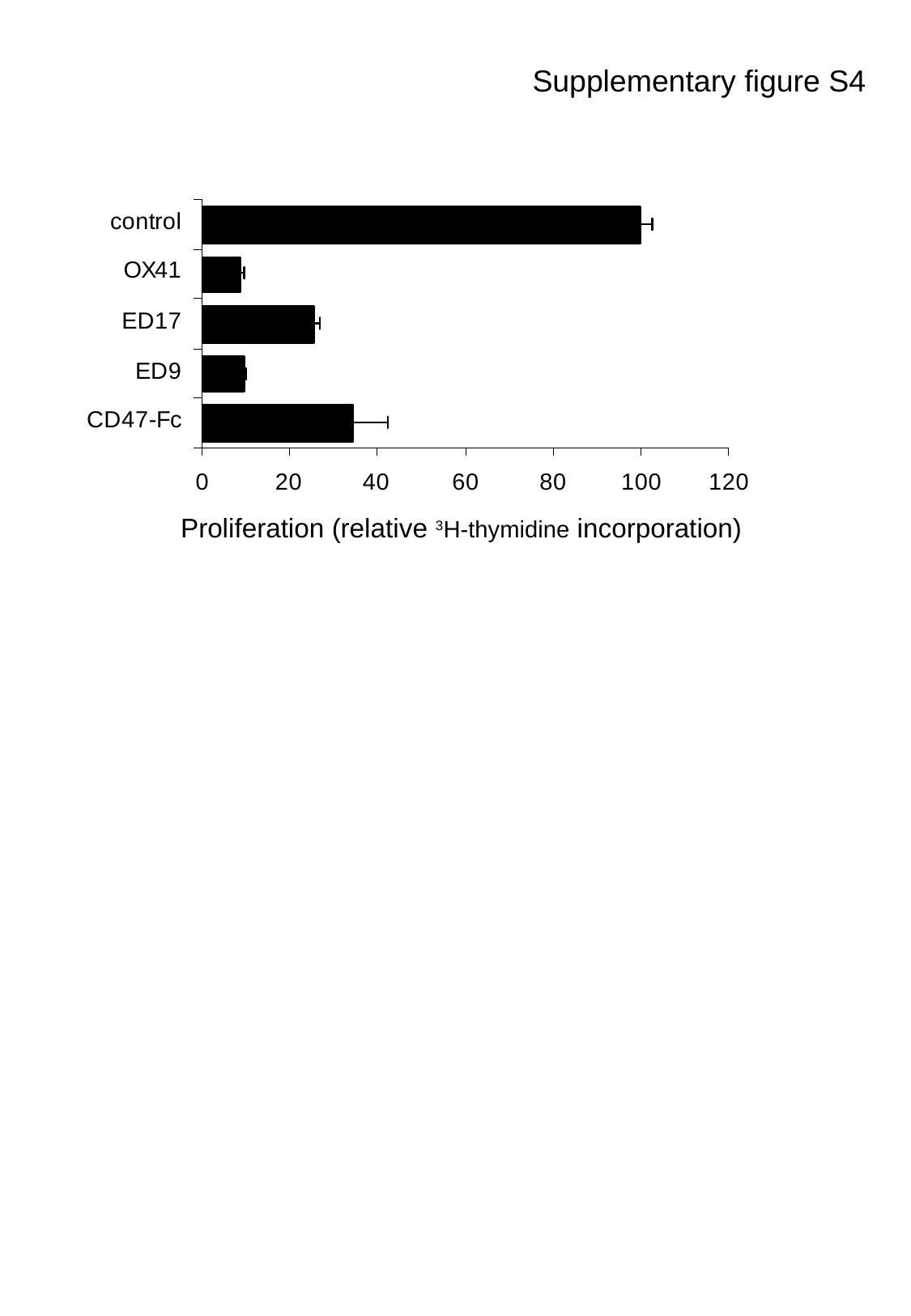

Supplementary figure S4
Proliferation (relative 3H-thymidine incorporation)

Supplement: Figure S4 — Triggering SIRPα in the rat NR8383 macrophage cell line inhibits proliferation. NR8383 cells were incubated for 18 hours with CD47-Fc protein or indicated anti-rat SIRPα monoclonal antibodies (ED9, ED17 or OX41). 3H-thymidine was added for 4 hours and proliferation was determined by incorporated radioactivity. (PPT) [file pone.0052143.s004.ppt]

## Slide 1
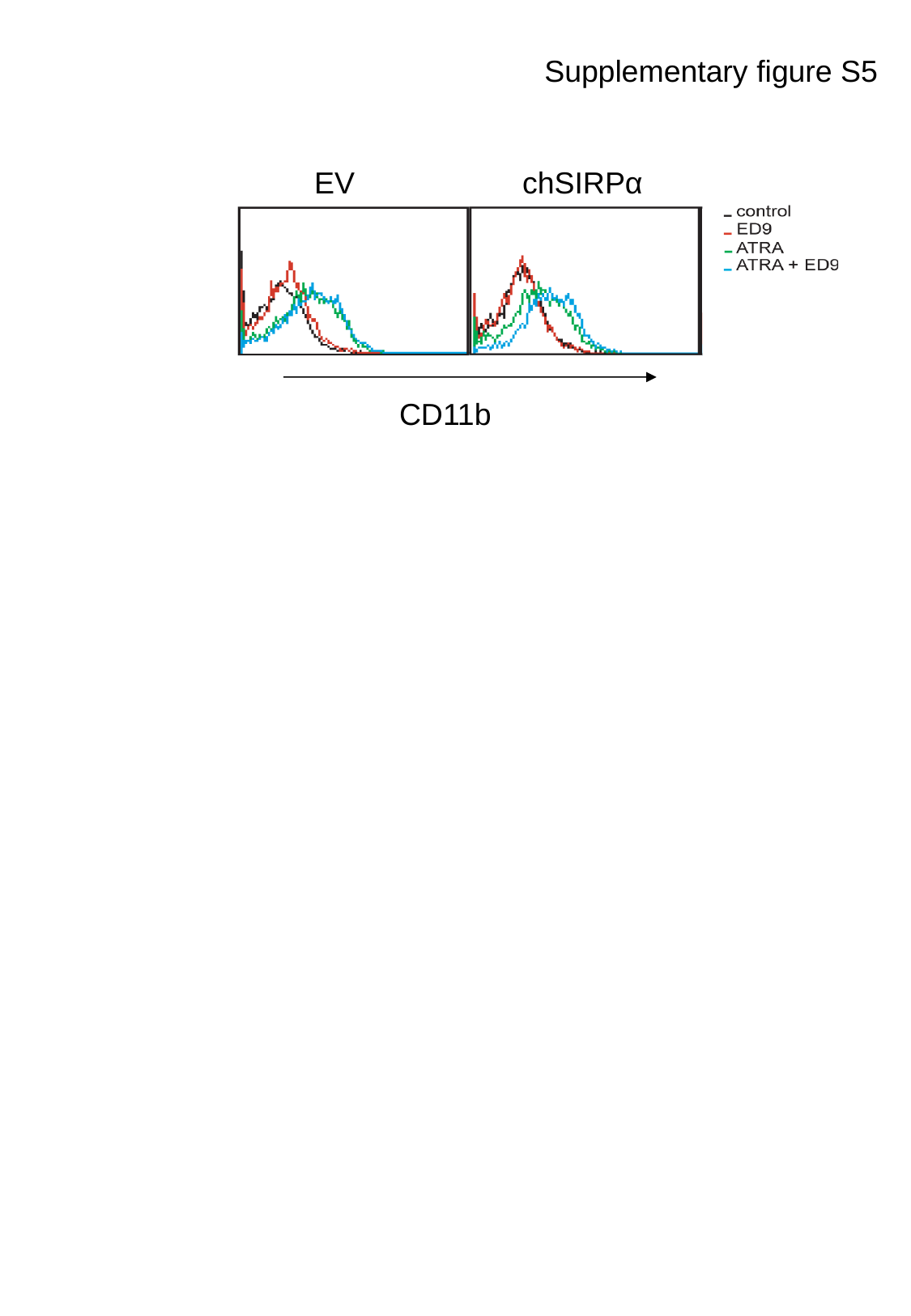

Supplementary figure S5
EV
chSIRPα
CD11b

Supplement: Figure S5 — NB4 cells differentiate by ATRA exposure. Differentiation of NB4 cells stably expressing chSIRPα and EV was examined by flow cytometry after treatment with ATRA or ED9. increased expression of CD11b was observed only after ATRA but not by ED9 treatment. (PPT) [file pone.0052143.s005.ppt]

## Slide 1
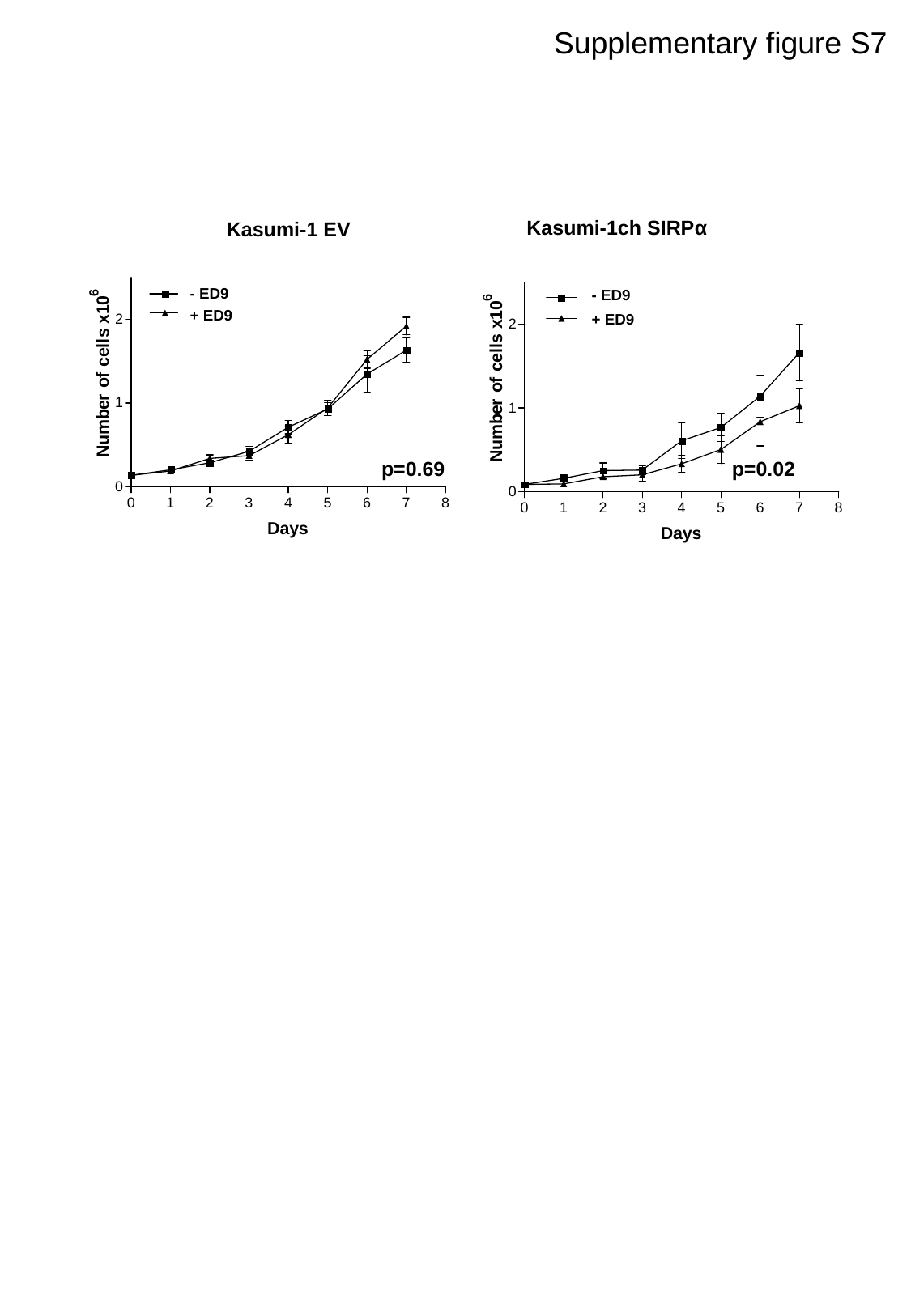

Supplementary figure S7
Kasumi-1ch SIRPα
Kasumi-1 EV
- ED9
+ ED9
- ED9
+ ED9
p=0.69
p=0.02

Supplement: Figure S7 — SIRPα ligation results in inhibition of proliferation in Kasumi-1 cells. Kasumi-1 cells expressing chSIRPα or EV, were incubated with ED9 mAb for 7 days and cell proliferation was evaluated by daily cell counting. Data are means ± SD calculated from 3 independent experiments using triplicate samples. (PPT) [file pone.0052143.s007.ppt]

## Slide 1
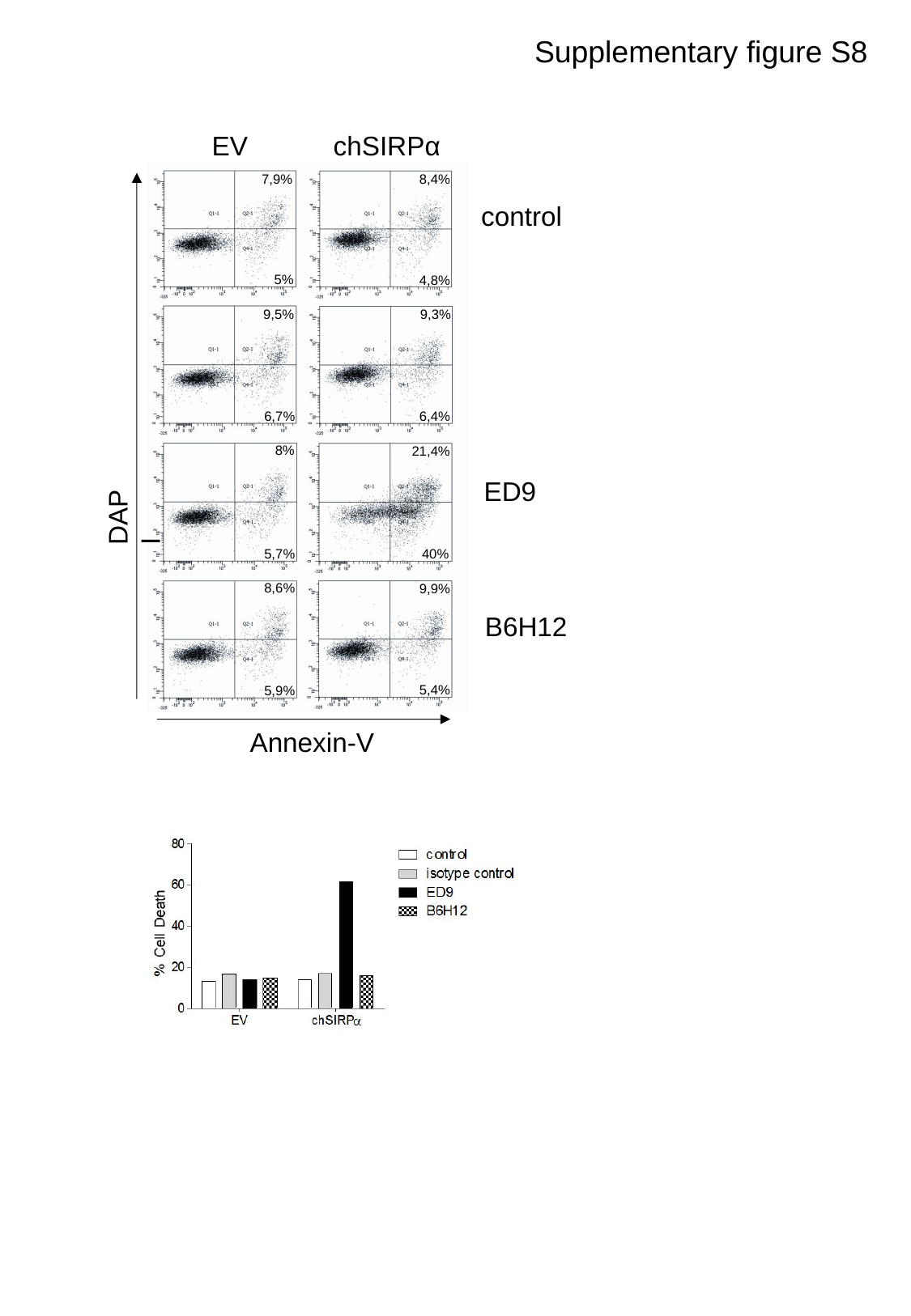

Supplementary figure S8
EV	chSIRPα
7,9%
8,4%
control
5%
4,8%
9,5%
9,3%
6,7%
6,4%
8%
21,4%
DAP
I
ED9
40%
5,7%
8,6%
9,9%
B6H12
5,4%
5,9%
Annexin-V

Supplement: Figure S8 — Blocking anti-CD47 antibody cannot mimic ED9 effects in Kasumi-1 cells. (A) Flow cytometry data of DAPI and Annexin-V staining and (B) Summary graph illustrates the quantified flow cytometric data. Kasumi-1 cells expressing chSIRPα or EV were incubated with ED9 mAb or B6H12 as blocking anti-CD47 antibody. Percentage of cell death was increased significantly in the case of ED9 treatment compared to EV but B6H12 anti-CD47 incubation did not have this effect. (PPT) [file pone.0052143.s008.ppt]
