# Supplementary material for: Engagement of SIRPα Inhibits Growth and Induces Programmed Cell Death in Acute Myeloid Leukemia Cells
Source: PLoS One. 2013 Jan 8;8(1):e52143. doi: 10.1371/journal.pone.0052143 (PMC3540026; doi:10.1371/journal.pone.0052143)
Supplement: Figure S2 — SIRPα mRNA expression in adult AML cohort. (A) SIRPα mRNA expression was determined in different FAB subtypes of 285 adult patients. The dots represent individual patients and the horizontal bar is the mean of the group (ND: not determined). (B) Adapted correlation view of the 16 unsupervised clusters (indicated on the left) of 285 adult AML specimens identified by mRNA profiling [33], including the expression levels of SIRPα using 3 independent probes on the right diagonal axes. SIRPα expression is high in clusters 5, 9 and 16, but low in most other clusters, including clusters 12 and 13, which contain almost exclusively t(15;17) and t(8;21) AML, respectively. (PPT) [file pone.0052143.s002.ppt]

## Slide 1
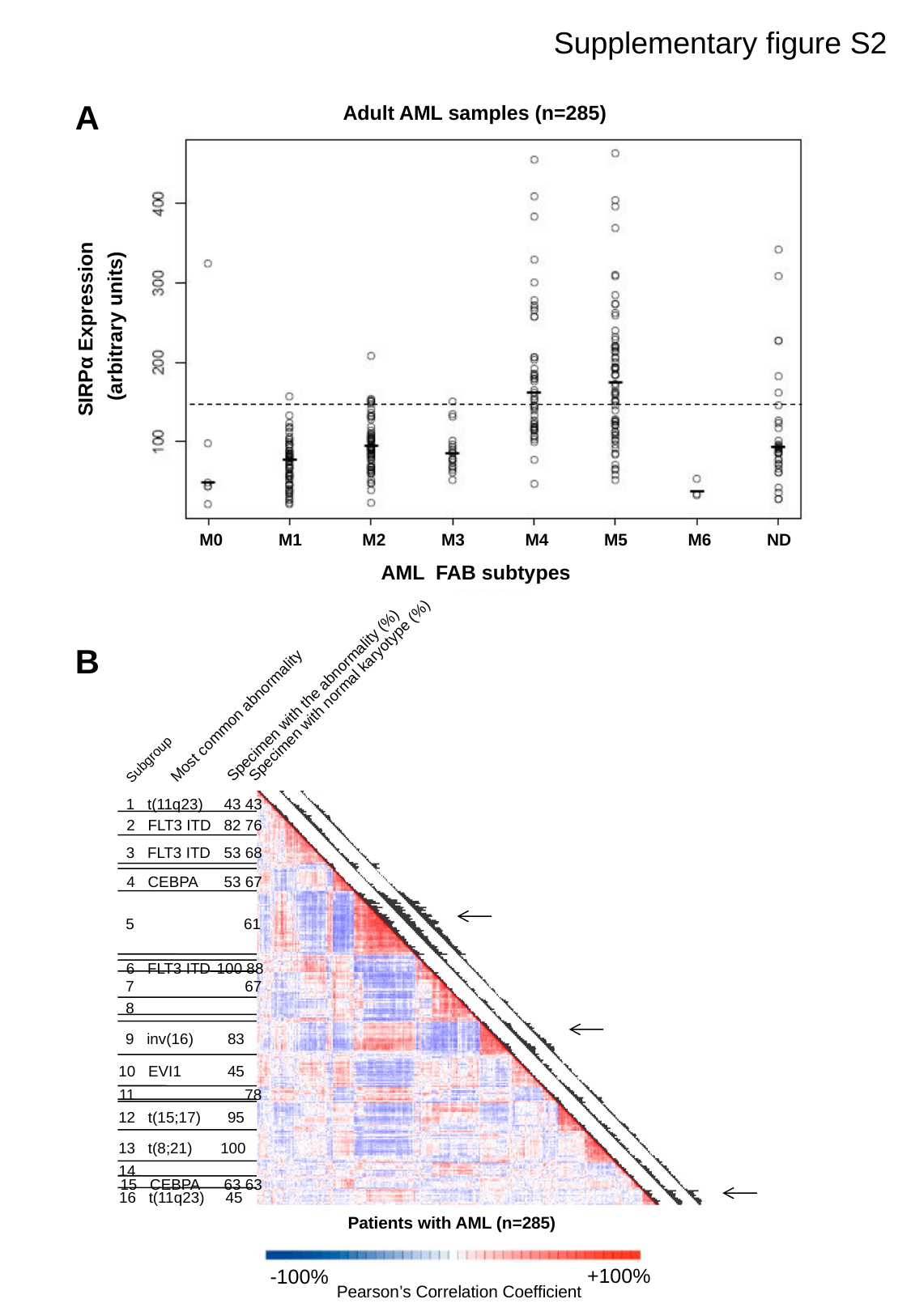

Supplementary figure S2
A
Adult AML samples (n=285)
SIRPα Expression
 (arbitrary units)
M0 M1 M2 M3 M4 M5 M6 ND
 AML FAB subtypes
Specimen with normal karyotype (%)
Specimen with the abnormality (%)
Most common abnormality
Subgroup
1 t(11q23)
43 43
2 FLT3 ITD
82 76
3 FLT3 ITD
53 68
4 CEBPA
53 67
5
 61
6 FLT3 ITD
100 88
7
 67
8
9 inv(16)
 83
10 EVI1
 45
11
 78
12 t(15;17)
 95
13 t(8;21)
 100
14
15 CEBPA
63 63
16 t(11q23)
 45
Patients with AML (n=285)
+100%
-100%
Pearson’s Correlation Coefficient
B
