# Supplementary material for: Engagement of SIRPα Inhibits Growth and Induces Programmed Cell Death in Acute Myeloid Leukemia Cells
Source: PLoS One. 2013 Jan 8;8(1):e52143. doi: 10.1371/journal.pone.0052143 (PMC3540026; doi:10.1371/journal.pone.0052143)
Supplement: Figure S6 — PTPNS1 promoter region is not methylated. Each circle indicates a CpG dinucleotide (open circles: unmethylated, filled circles: methylated) and each line represents analyses of a single amplified clone. SIRPα2p pseudogene, which is highly highly homologous to PTPNS1 was used as a positive control with high degree of methylation [49]. Methylation specific PCR and bisulphate sequencing [63] of the Kasumi-1 cell line and four t(8;21) AML patients did not reveal methylation of the PTPNS1 promoter region. (PPT) [file pone.0052143.s006.ppt]

## Slide 1
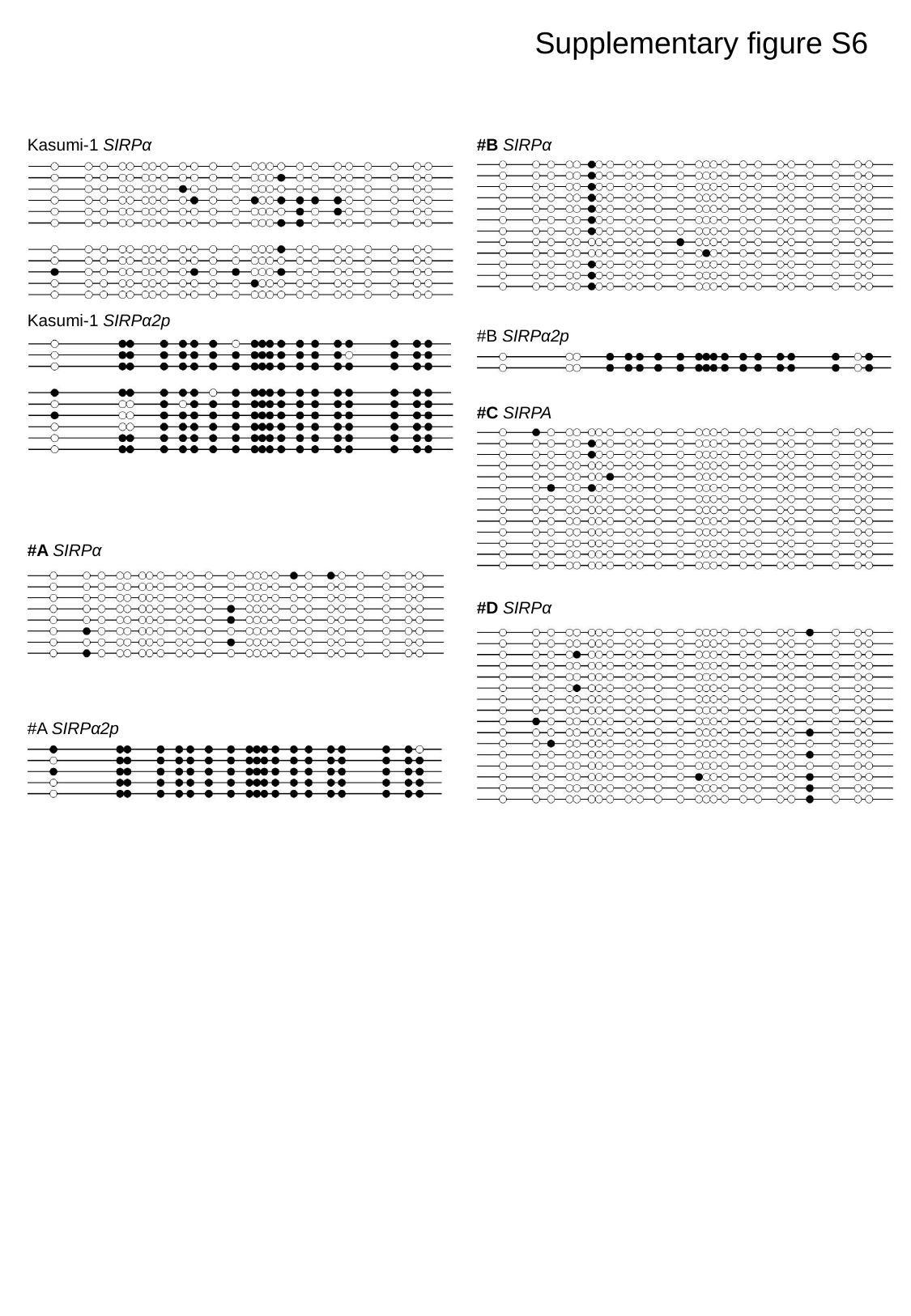

Supplementary figure S6
Kasumi-1 SIRPα
#B SIRPα
Kasumi-1 SIRPα2p
#B SIRPα2p
#C SIRPA
SIRP
SIRP
?
?
CpG
CpG
island
island
MSP2
MSP2
MSP1
MSP1
MSP Primers
MSP Primers
F1/R1
F1/R1
F3/R4
F3/R4
Sequence primers
Sequence primers
SIRP
SIRP
?
?
CpG
CpG
island
island
MSP2
MSP2
MSP1
MSP1
MSP Primers
MSP Primers
F1/R1
F1/R1
F3/R4
F3/R4
Sequence primers
Sequence primers
#A SIRPα
#D SIRPα
#A SIRPα2p
