# Supplementary material for: Engagement of SIRPα Inhibits Growth and Induces Programmed Cell Death in Acute Myeloid Leukemia Cells
Source: PLoS One. 2013 Jan 8;8(1):e52143. doi: 10.1371/journal.pone.0052143 (PMC3540026; doi:10.1371/journal.pone.0052143)
Supplement: Methods S1 — Detailed method description of the DNA bisulphate sequencing. (DOC) [file pone.0052143.s009.doc]

**Supplementary materials and methods**

***Bisulphite sequencing:*** Kasumi-1 cells and the 4 t(8;21) AML patient samples were bisulphate treated.[63] PCR amplification was performed using the following primer sets: *PTPNS1-F* 5’–GTATTTATTTTTAAGAGGGGTTTTTA-3’and *PTPNS1-R* 5’ CAAACTTATTTTTCTAAAATCAA-3’ in a two round PCR approach. PCR products were extracted from agarose gel by Qiagen gel-extraction kit (Qiagen Benelux BV) and cloned into the pGEM®-T Easy Vector Systems according to manufacturer’s protocol (Promega, Madison, WI, USA). After overnight incubation at 37°C clones were picked and cultured in liquid cultures at 37°C. The Qiagen DNA mini-prep kit (Qiagen Benelux BV) was used to extract DNA and the desired inserts were enzymatically cleaved from the pGEM®-T Easy Vector to check if the right insert was cloned. BaseClear BV performed automated DNA sequencing.
